# Pyoperitoneum Revealing a Spontaneous Renal Forniceal Rupture Due to Bladder Cancer: A Case Report

**DOI:** 10.7759/cureus.69976

**Published:** 2024-09-23

**Authors:** Atif Lifa, Anouar El Moudane, Oualid Bounouar, Ibrahim Boukhannous, Ali Barki

**Affiliations:** 1 Urology, Faculty of Medicine and Pharmacy of Oujda, Oujda, MAR; 2 Urology, Mohammed VI University Hospital, Oujda, MAR

**Keywords:** bladder cancer, forniceal rupture, infected ureterohydronephrosis, peritonitis, pyonephrosis

## Abstract

Extravasation of urine due to a forniceal rupture at the pelviureteric junction is a seldom-seen complication. While the presence of pyonephrosis can lead to a retroperitoneal abscess, its fistulization into the peritoneal cavity is exceptionally rare. Here, we present the case of a 50-year-old man exhibiting clinical signs of peritonitis. An abdominal CT scan indicated retroperitoneal peritonitis due to the rupture of the fornix. This case underscores the uncommon manifestation of pyonephrosis with peritonitis and pyoperitoneum, attributed to bladder cancer.

## Introduction

Renal fornix rupture is an uncommon progression of hydronephrosis resulting from urinary tract obstruction. While most instances are linked to ureteral stones, alternative causes have been documented, including external compression of the ureter, pregnancy, posterior urethral valve, intravenous fluid infusion, pyelo-ureteric junction syndrome, and, rarely, bladder outlet obstruction such as bladder cancer [1].

Bladder cancer ranks as the fourth most common cancer in terms of incidence and seventh in cancer-related mortality globally, making it the second most common urological cancer after prostate cancer [2]. It can cause various complications, such as weight loss, fatigue, urinary tract infections, metastasis, and urinary tract obstruction, which can potentially lead to chronic kidney failure [3].

Hydronephrosis, when complicated by infection, can lead to the development of pyonephrosis, which extends into the perirenal space and the psoas muscle. Although exceedingly rare, intraperitoneal fistulization can occur and may lead to a presentation of generalized peritonitis.

## Case presentation

A 50-year-old male patient, who had been a chronic active smoker for 15 years and had experienced intermittent, unexplored hematuria over the past month, was admitted to the emergency department due to diffuse abdominal pain that began in the left lumbar region three weeks prior. On clinical assessment, he was conscious, hemodynamically, and respiratorily stable, with a fever of 39.2°C. The conjunctiva appeared normal-colored, and there was diffuse abdominal rigidity. A digital rectal examination revealed a prostate estimated to weigh 30 g.

The biological assessment revealed an infectious syndrome, with a C-reactive protein level of 100 mg/L and an elevated white blood cell count of 19,850/mm³. Hemoglobin was measured at 12 g/dL, platelets at 453,000/mm³, and there was a slight impairment in renal function, indicated by a creatinine level of 15 mg/L and urea at 0.4 g/L, while the blood ionogram remained normal (Table 1).

**Table 1:** Laboratory results.

Results	Value	Normal value
C-reactive protein	100 mg/L	<6 mg/L
White blood cell	19,850/mm³	3,000–10,000/mm³
Hemoglobin	12 g/dL	13–18 g/dL
Platelets	453,000 /mm³	150,000–450,000/mm^3^
Urea	0.4 g/L	<0.5 g/L
Creatinine level	15 mg/L	<12 mg/L

The urine analysis (cytobacteriological examination of urine) showed leukocyturia at 340,000 and hematuria at 190,000. However, the urine culture was sterile.

**Figure 1:**
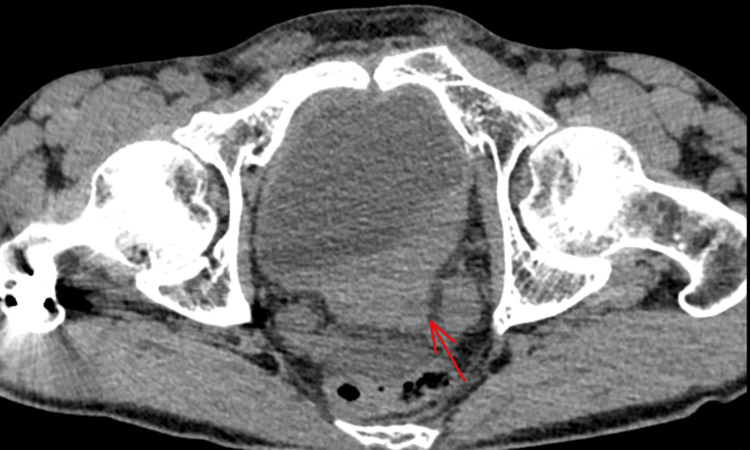
Axial section of a CT of the abdomen presenting a tumor of the left posterolateral wall of the bladder (red arrow).

**Figure 2:**
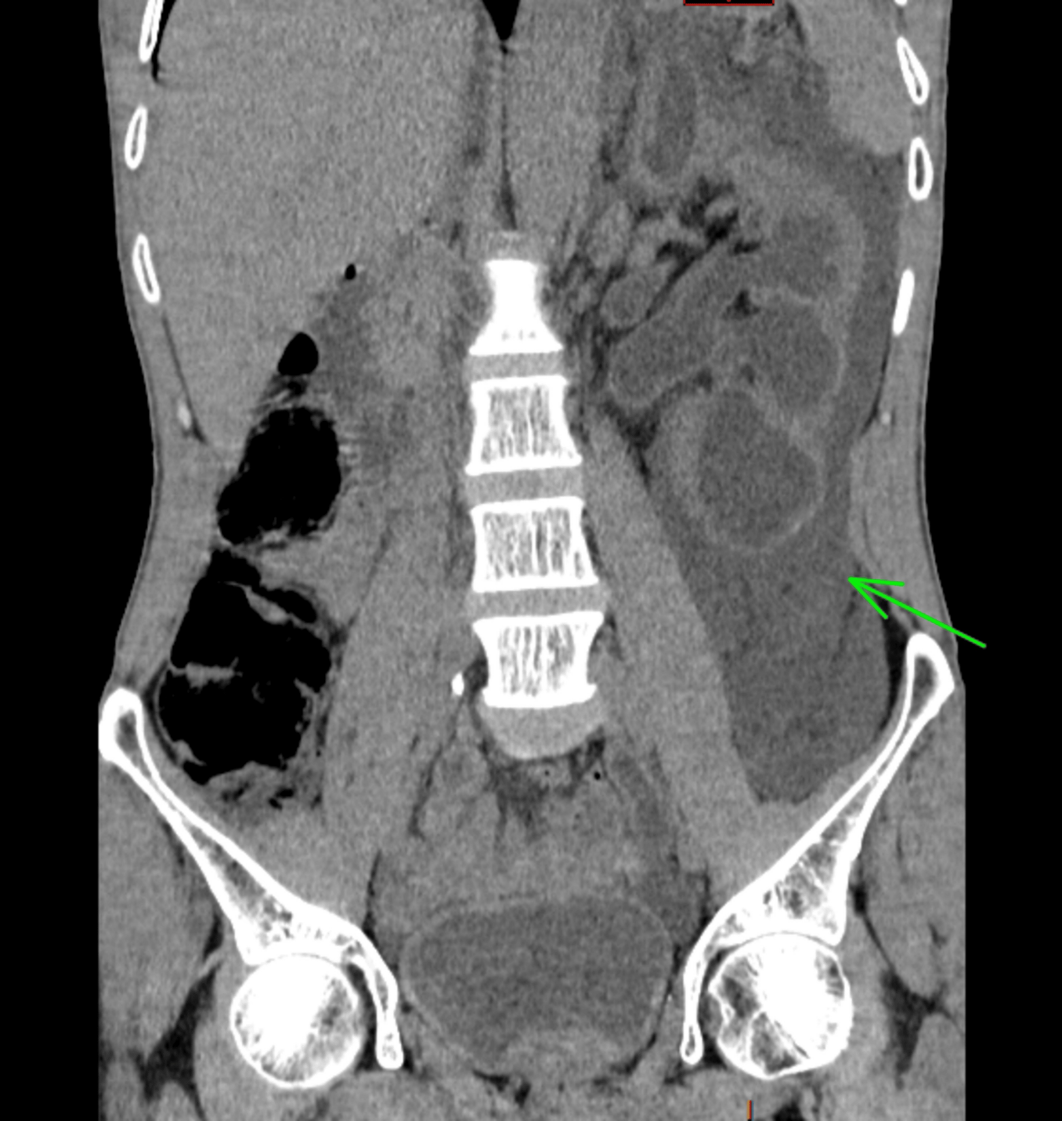
Coronal section of a CT of the abdomen revealing perinephric collection (green arrow).

**Figure 3:**
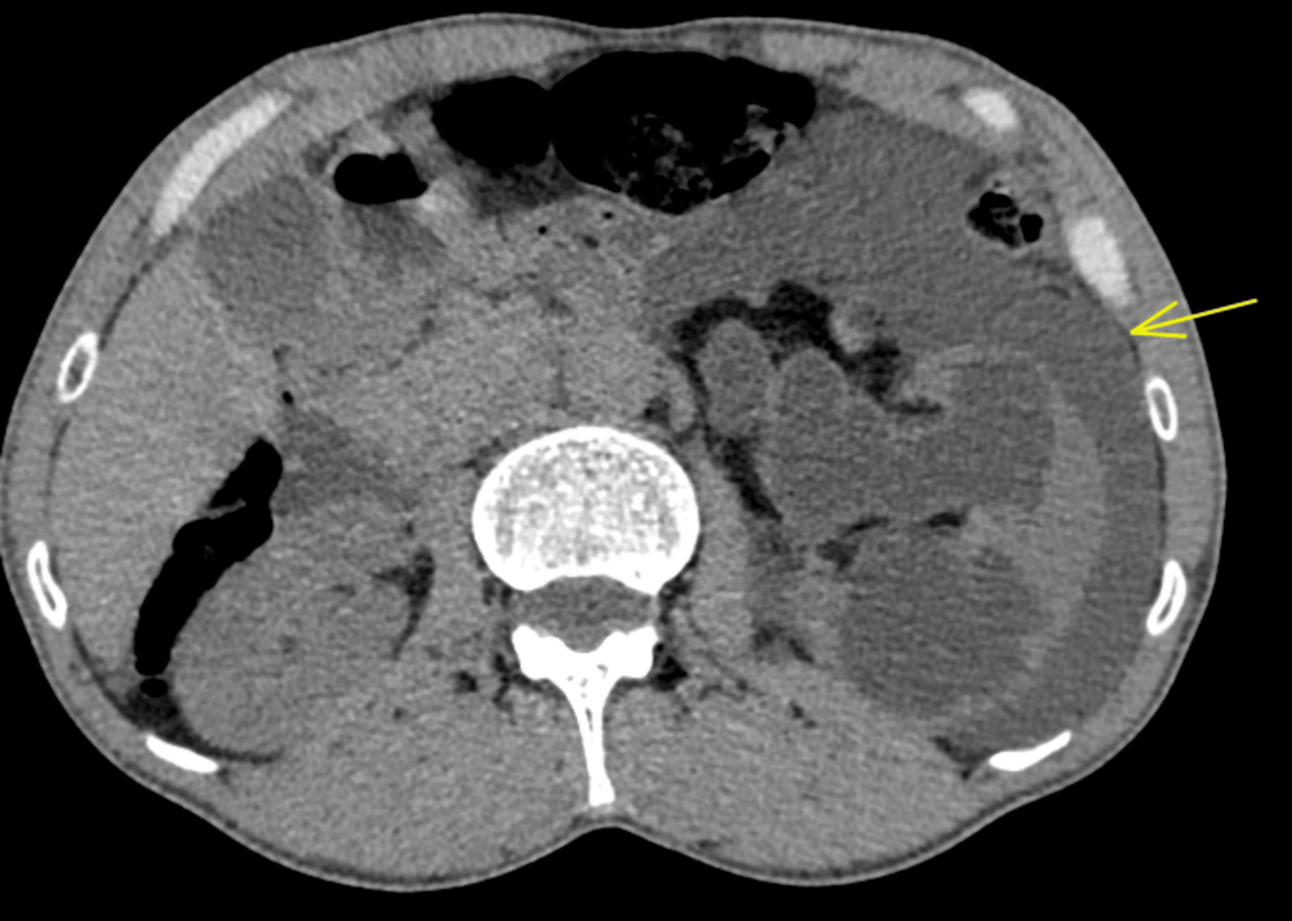
Axial section of a CT of the abdomen revealing a significant left pyelo-ureteral dilatation with perinephric collection (yellow arrow).

A contrast-enhanced abdominopelvic CT scan revealed a tumor on the left posterolateral wall of the bladder (Figure 1), which was responsible for ureterohydronephrosis with urinoma and intraperitoneal fluid accumulation, likely due to a rupture of the fornix (Figures 2, 3).

**Figure 4:**
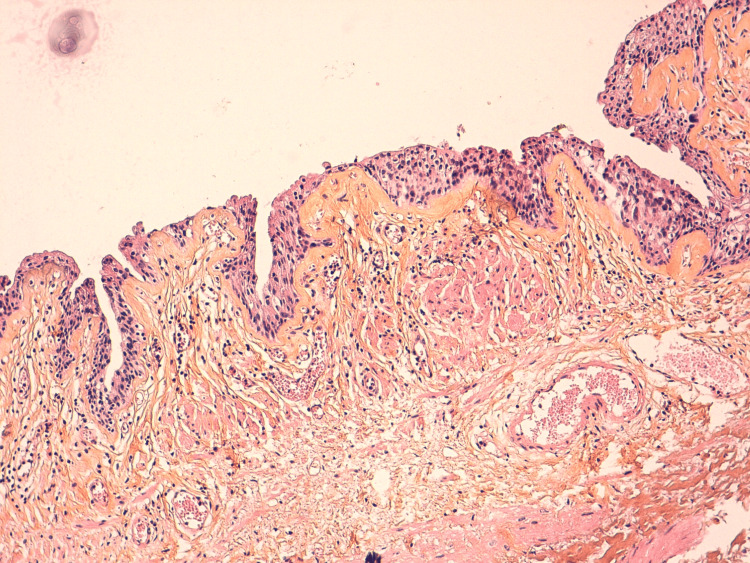
Histological findings (hematoxylin and eosin ×10). A histological section shows an urothelial lining that is abraded in some areas, displaying architectural disorganization. The cells exhibit moderate cytological atypia and mitoses are confined to the lower two-thirds of the lining. This proliferation does not exceed the basement membrane and does not infiltrate the stroma (hematoxylin and eosin ×10).

The patient was managed with emergency hospitalization, followed by transurethral resection of the bladder tumor and an attempt to place a double-J stent. Cystoscopy confirmed the presence of a tumor on the left wall of the bladder. Transurethral resection of the bladder tumor was performed, and although the left ureteral orifice was difficult to identify, a double-J stent was successfully placed. He also received broad-spectrum antibiotics and was carefully surveilled. Histological examination indicated urothelial carcinoma in situ, with no muscle invasion (Figure 4).

The postoperative course was uneventful. The pain disappeared and the renal function returned to normal.

## Discussion

Fornix rupture, a seldom encountered condition, is typically detected radiologically due to urinary extravasation around the kidney resulting from ureteral obstruction. The leading cause of this rupture is ureteral stones, although other factors such as malignant compression of the ureter, pregnancy, posterior urethral valves, external vascular compression, iatrogenic, or idiopathic factors can also contribute, albeit rarely [1]. In the case discussed, the ureterohydronephrosis leading to fornix rupture was secondary to a bladder tumor.

Bladder cancer can lead to urinary tract obstruction in several ways. As the tumor grows, it may invade or compress surrounding structures, including the ureters. This compression or invasion can obstruct the normal flow of urine, causing hydronephrosis. When the obstruction is severe and persistent, the increased pressure within the renal pelvis can result in a forniceal rupture. This rupture allows urine to leak into the surrounding retroperitoneal space, potentially leading to pyonephrosis [4].

Pyoperitoneum and peritonitis often stem from intraperitoneal issues, typically related to digestive conditions such as gallstones or pancreatitis. While peritoneal irritation resulting from pyelonephritis can sometimes trigger peritonitis, the prognosis is usually favorable. However, instances of pyonephrosis rupture causing intraperitoneal collection are rarely documented.

Elmoudane et al. recently documented a case where a ruptured pyonephrosis resulted in peritonitis in a patient with a damaged kidney at the pelvic-ureteral junction, which was managed through nephrectomy [5]. Larrache et al. reported a case of spontaneous renal forniceal rupture due to a bladder tumor in 2020 [6]. Hendaoui et al. reported a case in 1999 where intraperitoneal extension occurred in a patient with a non-functioning kidney due to urolithiasis [7]. Boulay et al. reported a rare case of spontaneous renal forniceal rupture secondary to pelvic inflammatory disease [8]. Another case involved spontaneous rupture of pyonephrosis leading to pyoperitoneum, even in the absence of urinary tract obstruction [4].

## Conclusions

This case highlights a rare complication of bladder cancer, where ureterohydronephrosis led to a forniceal rupture, resulting in retroperitoneal peritonitis and pyoperitoneum. This unusual presentation underscores the importance of early detection and management of urinary obstructions, particularly in patients with malignancies. Prompt intervention, including imaging and surgical management, is crucial in preventing severe outcomes such as renal failure or widespread infection. The primary treatment involves immediate urinary diversion, followed by addressing the underlying pathology.
